# Who Returns to work? Exploring the Role of Interpersonal Problems in Occupational Rehabilitation

**DOI:** 10.1007/s10926-022-10091-2

**Published:** 2023-06-20

**Authors:** David Aleksander Nilsen, Oda Nissen, Trond Nordfjærn, Karen Walseth Hara, Tore C. Stiles

**Affiliations:** 1 St. Olav’s University Hospital, Trondheim, Norway; 2 Department of Psychology, Faculty of Social and Educational Sciences, Norwegian University of Science and Technology (NTNU), Trondheim, Norway; 3 Department of Public Health and Nursing, Faculty of Medicine and Health Sciences, Norwegian University of Science and Technology (NTNU), Trondheim, Norway

**Keywords:** Return to work, Personality, Interpersonal psychology, Interpersonal problems, Hostile-dominance, Occupational rehabilitation

## Abstract

**Purpose:** Current interventions designed to facilitate return to work (RTW) after long-term sick leave show weak effects, indicating the need for new approaches to the RTW process. The importance of social relationships in the workplace is widely recognized in existing RTW literature, but very little is known about the role of the *interpersonal problems* of the returning worker. Current research indicates that a subset of these – *hostile-dominant interpersonal problems* – give particular disadvantages in several life areas. This prospective cohort study aims to test whether higher levels of interpersonal problems predict lower likelihood of RTW when controlling for symptom levels (H1); and whether higher levels of hostile-dominant interpersonal problems specifically predict lower likelihood of RTW (H2). **Methods:** 189 patients on long-term sick leave completed a 3½-week transdiagnostic RTW program. Before treatment, self-reported interpersonal problems, chronic pain, insomnia, fatigue levels, anxiety and depression were collected. RTW data for the following year were collected from the Norwegian Labour and Welfare Administration. **Results:** A multivariable binary logistic regression analysis found that (H2) hostile-dominant interpersonal problems significantly predicted RTW (OR = 0.44, [95% CI: 0.19, 0.98], *p* = .045), while a corresponding analysis (H1) found that general interpersonal problems did not. **Conclusion:** Hostile-dominant interpersonal problems negatively predict RTW after long-term sick leave, suggesting an overlooked factor in the field of occupational rehabilitation. The findings could open up new avenues for research and interventions entailed to individuals in the field of occupational rehabilitation.

## Introduction

Long-term sick leave incurs significant economic and social costs on individuals, families, workplaces and society at large [1]. Facilitating effective and lasting return to work (RTW) after long-term sick leave is the primary goal in the field of occupational rehabilitation. A particular challenge in this field is helping patients with *subjective health complaints*, characterized by symptoms with no clear biological markers, and where medical investigation offers few objective findings [2]. The main groups of subjective health complaints – musculoskeletal and mental disorders – are the cause of the majority of both sick leave cases and sick leave days in Norway [3].

Recent decades has seen a gradual shift from a biomedical to a biopsychosocial understanding of disability [see 4]. Instead of a narrow focus on individual medical impairment, we now consider the interactions between medical, psychological, psychosocial and system-based factors to understand and improve the RTW process [5]. This shift encourages transdiagnostic explanations and interventions, which is of particular interest when treating subjective health complaints, where diagnoses are of questionable utility [6]. For these patients, comorbidity is the rule rather than the exception – both when it comes to overall comorbidity [see 7] and comorbidity between mental disorders [see 8]. Comorbidity does not necessarily reflect multiple underlying problems – it could also indicate that one is investigating the problem at the wrong level [8].

Symptom improvement was once thought to be a necessary and sufficient prerequisite of RTW. Recent meta-analyses strongly challenge this view, indicating that symptom improvement plays a rather minor role in this process, particularly in the case of subjective health complaints [9, 10]. Additionally, symptom improvement often follows RTW instead of preceding it [11, 12]. It seems abundantly clear that symptom improvement is not the solution to the RTW puzzle - other factors are deciding who returns to work.

In the last couple of decades, an extensive research effort has been made to identify these factors affecting RTW. While numerous factors have been identified, the literature stresses that the psychosocial domain has received relatively little attention compared to, for example, research on workplace accommodations [13]. Two particular areas of the psychosocial domain are singled out as being under-studied, yet important: Individual traits (i.e. personality factors) [14], and social relations in the workplace [15]. The present study will address both individual traits and social relations, by focusing on the area where these two overlap: *Interpersonal problems*, the core concept within the broader field of interpersonal psychology.

Interpersonal problems are relatively stable individual traits that affect the ability to build and maintain healthy relationships. The problems can be differentiated along underlying dimensions of dominance vs. submissiveness and hostility vs. friendliness [16–18].

All types of interpersonal problems are frequently reported by psychiatric patients [17, 19, 20]. They are seen as the core pathology in the broader concept of personality disorders, and significant contributors to the incidence, prevalence and course of other mental health disorders [19, 21, 22]. Personality disorders are characterized by impairment in aspects of the self (e.g. identity, self-direction and self-worth) and interpersonal functioning (i.e. interpersonal problems) that has persisted over a longer period of time and is associated with significant distress or impairment. [23] Interpersonal problems can be reduced in psychotherapy [24, 25], making them modifiable factors, and therefore of particular research interest.

Mental disorders and other health complaints also affect our interactions with others, and may create secondary, state-dependent (i.e. temporary) interpersonal problems. People who are depressed, fatigued or in pain are shown to have higher levels of interpersonal problems for the duration of the symptoms [26], for example manifesting as neediness and irritability.

While associations between personality disorders and unemployment have been shown [see 27], the role of interpersonal problems in the process of returning to work after long term sick leave has received little to no attention in the literature. Interpersonal problems are generally poorly investigated outside of the field of clinical psychology, which is surprising considering their impact on social functioning and the importance of social relations in all domains of life.

There are several reasons to examine the connection between interpersonal problems and RTW in particular. First, recent literature on RTW stresses the importance of good social relations in the workplace, and how it is an important predictor of successful and enduring RTW [15]. A review of interventions aimed at facilitating good relationships in the workplace [28] include “sensitivity training” for employers and co-workers, but curiously mentions no equivalent interventions targeting the returning worker.

Second, the importance of individual differences in personality should not be underestimated. Dysfunctional personality traits are associated with conflict at work, being laid off, and receiving disability pension [27, 29]. Returning to work is a “complex behavior in which individual perceptions, beliefs and decisions are crucial” [14]. In an RTW context, most of these perceptions, beliefs and decisions will involve other people in some way – and interpersonal psychology can help us understand individual differences in this area.

Third, holding a job means cooperating and interacting with others. The most detailed knowledge available on the mechanisms of human connection and cooperation is arguably found in the field of psychotherapy research. In this tradition, the *therapeutic alliance* is a core term, referring to the bond of trust and cooperation between therapist and client [30]. Independently of the theoretical framework or type of treatment used, the therapeutic alliance consistently appears as the most robust predictor of outcome of therapy [31–33], and an important predictor of the quality of this alliance is the interpersonal problems of the patient [33, 34]. Crucially, the phenomenon of alliance is not limited to therapeutic relationships – it influences every relationship, including those in the workplace. These arguments form the rationale for our first hypothesis (H1).

Our second hypothesis (H2) concerns a specific subtype of interpersonal problems, characterized by high hostility and dominance. These styles seem to have particularly adverse outcomes on relationships [35], and might, therefore, be particularly detrimental to successful RTW. People with high levels of hostile-dominant interpersonal problems (HDIP) are typically characterized as controlling, suspicious, vindictive and cold, which can provoke inferiority, distrust and uncertainty in others [16, 18]. Paranoid, narcissistic and dyssocial personality disorders are characterised by severe HDIP [19, 35], and are known to cause more problems in the workplace than other personality disorders [36]. In psychotherapy research, where HDIP have been studied the most, patients with higher levels of HDIP tend to have a poorer therapeutic alliance than patients with other interpersonal problems, resulting in poorer outcome of therapy [37–39].

Hostile and dominant styles are, almost by definition, linked to interpersonal conflict and hostility, the logical opposite of good relationships. Workplace conflict is a major and growing problem in organizations, and is a strong predictor of stress, burnout, subjective health complaints and eventual sick leave [40, 41]. For these reasons, we expect HDIP in particular to impede RTW.

Special care needs to be taken when investigating the contributions of interpersonal problems on long-term outcomes like RTW, since measures of the construct are sensitive to both trait-dependent and state-dependent interpersonal problems [26, 42]. Since levels of interpersonal problems are influenced by subjective health complaints at the time of measurement, these must be controlled for in order to eliminate state-dependent fluctuations.

This paper aims to explore the connection between interpersonal problems and RTW, using data from a randomized controlled trial involving a 3.5-week transdiagnostic occupational rehabilitation program (see [43] for study protocol). Specifically, the following hypotheses will be tested:

1. Higher levels of interpersonal problems predict a lower likelihood of RTW when controlling for symptom levels.
2. Higher levels of hostile-dominant interpersonal problems specifically predict such an lower likelihood of RTW.

## Method

### Design and Procedures

This prospective cohort study is based on data from a Norwegian research project on occupational rehabilitation, which was conducted at Hysnes Occupational Rehabilitation Center, St. Olav’s University Hospital in Norway. The details of this randomized controlled trial, including context, funding, recruitment procedure and rehabilitation interventions, is described in the study protocol [43].

Patients were referred to the occupational rehabilitation programme by their general practitioner between January 2012 and June 2013, and invited to participate in the study upon admission. The inclusion criteria required participants to be 18–60 years of age, and that they had been on sick leave for at least eight weeks due to musculoskeletal disorders, pain, fatigue and/or common mental disorders. They had to have a self-defined goal of increasing work participation. They also had to be able to speak Norwegian, be able to attend rehabilitation between 8:30 A.M. and 3:00 P.M. on weekdays, and be able to maintain basic care for themselves while at the clinic. Exclusion criteria were pregnancy, severe mental illness (ongoing mania, psychosis or suicidal ideation), substance abuse and addiction. Moreover, patients had to have been assessed and treated for any physical health problems before being admitted to the clinic. All participants were evaluated according to the inclusion criteria by a multidisciplinary team consisting of a psychologist, a physician and a physiotherapist.

Before inclusion in the study, all the referred patients were asked to complete an online self-report survey consisting of 18 different questionnaires (386 items in total). Norwegian versions of the forms were filled out through CheckWare, an online tool for self-report questionnaires. The population is the same that was included in a recent study by Hara et al. [44], which provide a more detailed description of the participants, including employment status and type of sickness benefits received. Pre-rehabilitation, 36% of the patients were on partial sick leave and the rest on full-time sick leave, and 60% in total were employed. Of temporary medical benefits, 57% received work assessment allowance and the remaining 43% received sickness benefits.

All participants completed a 3.5 week (17 six to seven-hour days) occupational rehabilitation programme. The rehabilitation was a mix of individual and group-based interventions, with a maximum of eight participants in each group. The core element of the rehabilitation was based on Acceptance and Commitment Therapy (ACT), and three main areas were targeted: mental training, physical training and work-related problem-solving. Return to work (RTW) data were collected by the Norwegian Labour and Welfare Service (NAV) in the year following rehabilitation.

### Assessments

**Interpersonal problems.** Interpersonal problems were assessed using a 64-item version of the Inventory of Interpersonal Problems, IIP-64 [18]. The IIP-64 is a self-report questionnaire consisting of 64 items. The respondent rates interpersonal behaviour which is either “hard for me to do” or that “you do too much” on a 5-point Likert scale rating from 0 (“Not at all”) to 4 (“Extremely”). The scores weigh on eight subscales that have a circumplex relation to one another: Domineering, Vindictive, Cold, Socially Avoidant, Non-assertive, Exploitable, Overly Nurturant, and Intrusive.

A global IIP-64 score (IP) was calculated as the average of the eight subscales, indicating the overall level of IP experienced (range: 0.0–4.0). Hostile-dominant interpersonal problems (HDIP) was calculated from the subscales that make up the hostile-dominant quadrant of the interpersonal circumplex, using the method described in Ruiz et al. [45]: HDIP = Vindictive + (0.707 * Domineering) + (0.707 * Cold). This calculation yields total HDIP scores ranging from 0.0 to 9.66. For purposes of comparison with overall IP, these values have been scaled down to also fit within a range of 0.0–4.0. The internal consistency of the scales has been found to be good both in American and Norwegian samples [18, 46]. In the current study, Cronbach’s alpha for IP and HDIP were 0.96 and 0.89 respectively, and average inter-item correlation (AIIC) was 0.26 for IP and 0.28 for HDIP.

**Anxiety and depression.** Levels of anxiety and depression were measured using the Hospital Anxiety and Depression Scale (HADS) [47]. The scale consists of 14 items (α = 0.88, AAIC = 0.35) about how the respondent has been feeling the last week, with half weighing on anxiety and half on depression. Items are rated on a 4-point Likert-scale (0–3). A total HADS score, indicating the overall level of depression and anxiety, was used as a continuous variable in the analyses. A score of 8 or more on either subscale indicates borderline or abnormal levels of anxiety or depression, but is not equal to clinical diagnoses. The psychometric properties of the scale have been reported as good, and good internal consistency was also found in studies on Norwegian populations using the Norwegian translation of HADS [48].

**Fatigue.** To assess levels of fatigue, Chalder Fatigue Scale (CFS) was used. The scale consists of 11 items (α = 0.87, AIIC = 0.38) about problems the respondent has had with feeling tired, weak or lacking in energy in the last month. It covers both physical and mental fatigue. Answers are scored bimodally (0-0-1-1), giving total scores from 0 to 11. Scores of 4 or greater are considered clinical caseness. The scale has high reliability and validity scores [49].

**Insomnia.** Sleep problems were measured with the Insomnia Severity Index (ISI), which consists of 7 items, (α = 0.86, AIIC = 0.35) assessing level of sleep difficulties, to what degree these difficulties reduce function and quality of life, how worried the respondent is about the problems and how visible he perceives them to be for others. Answers are given on 5-point Likert scales (0–4), yielding results from 0 to 28 with a clinical cutoff at 15 points. The measure has been found to have adequate internal consistency and is recommended as a standard measure of insomnia symptoms [50].

**Chronic pain.** Level of chronic pain was assessed using one item from the Short Form-8 health survey (SF-8), asking “How much bodily pain have you had during the last week? (no pain, very mild, mild, moderate, severe or very severe)”. The use of this single recall item to measure chronic pain has been validated in a large Norwegian sample from a population study (the HUNT 3 study), using a clinical cutoff at moderate pain [51].

**Outcome measure: Return to work (RTW).** Work participation following rehabilitation was retrieved from the database of the Norwegian Labour and Welfare Administration (NAV). The total number of paid work hours per week was collected biweekly from 6 weeks prior to entering the rehabilitation and up to 58 weeks after completion, and data from the year (52 weeks) following rehabilitation was used.

Hara et al. [52] argue that participation in competitive work one day (7.5 h) or more per week represents a meaningful first step towards entering the ordinary workforce. The present study has applied this cutoff in the same manner: participants were dichotomized into groups based on whether they on average were able to work for at least one day a week during the first year following rehabilitation (*n* = 90), or not (*n* = 99).

### Statistical Procedures

Data analyses were performed using IBM SPSS statistics, version 25 (https://www.ibm.com/spss). From the original dataset of 212 cases, 23 were excluded (*N* = 189) for missing the majority of data from the main measurements, IIP-64 or RTW. Independent-samples t-tests were performed to investigate whether the excluded participants differed from the remaining sample on any measured variables.

Cronbach’s alpha was calculated to assess the internal validity of the scales. Because scales with many items might cause positive inflation of the alpha value, average inter-item correlation (AIIC, using Pearson’s *r* correlations) was calculated as an additional indication of internal consistency. Cronbach’s alpha values above 0.70 and AIIC between 0.15 and 0.50 are considered indications of good internal consistency [53, 54].

Descriptive statistics were used to describe the population at baseline, and independent samples t-tests were used to compare the group that did not return to work with the one that did. Cohens *d* was calculated to estimate effect sizes of differences between means, and Pearson’s *r* correlation coefficients were calculated to assess correlations between the main continuous variables (Table 2) [55].

To test whether IP or HDIP predicted RTW, two multivariable binary logistic regression analyses were used. Two separate models were used to avoid issues of collinearity due to overlap between the HDIP and IP constructs. In both analyses, the following variables were treated as covariates: age, gender, pain, insomnia, fatigue, anxiety/depression. All variables except gender were treated as continuous and were entered simultaneously, as their individual contributions are irrelevant to our hypothesis. A conventional p-value of 0.05 was employed to evaluate statistical significance.

## Results

### Sample Characteristics

A total of 189 participants were included in the study, with a mean age of 43 years (range 22 to 61). A majority were women (80%, *n* = 151). At intake, 75% of the participants reported chronic pain, 34% had significant sleep problems, 92% were fatigued, and 61% had at least borderline levels of anxiety, depression or both (32%). This reflects high levels of comorbidity between clinical symptoms, but should not be mistaken for clinical diagnoses. Symptom levels and interpersonal problems for the participants who did and did not return to work are reported in Table 1. There were no statistically significant differences between the two groups on any of the measures.

**Table 1:** Descriptive statistics for all participants, and comparisons of participants who did and did not RTW.

	Total sample (*N* = 189)			Did not RTW (*n* = 99)			Did RTW (*n* = 90)			*t*-test		
Variable	*M* / %	*SD* / (*n*)		*M* / %	*SD* / (*n*)		*M* / %	*SD* / (*n*)		*t*	*p*	*D*
Age	43.12	9.11		42.09	9.38		44.26	8.72		-1.64	0.10	0.24
Gender (female)	80%	(151)		76%	(75)		84%	(76)		-1.49	0.14	0.20
Pain	3.90	1.16		4.01	1.14		3.78	1.18		1.38	0.17	0.20
Insomnia	11.83	6.26		11.75	6.14		11.91	6.43		-0.18	0.86	0.03
Fatigue	8.27	2.67		8.23	2.59		8.31	2.77		-0.84	0.40	0.03
Anxiety/Depression	15.00	7.43		14.57	7.77		15.48	7.06		-0.20	0.84	0.12
IP	0.94	0.53		0.92	0.57		0.96	0.49		-0.43	0.67	0.07
HDIP	0.53	0.47		0.57	0.51		0.49	0.41		1.07	0.28	0.11
RTW	9.74	10.69		1.11	1.76		19.23	8.00		-^a^	-^a^	-^a^

*Note.* Pain = score on level of somatic pain from the Short-Form Health Status-8. Fatigue = sum score on the Chalder Fatigue Scale. Insomnia = sum score on the Insomnia Severity Index. Anxiety/Depression = sum score on the Hospital Anxiety and Depression Scale. IP = sum score on the Inventory of Interpersonal Problems. HDIP = calculated sub-score from the Inventory of Interpersonal Problems. RTW (return to work) = average hours worked per week in the first year after rehabilitation. All elements represent raw, nonstandardized scores. ^a^Score is redundant because this measure was used to split the groups

### Analyses and Hypothesis Testing

No significant differences were found between the included and excluded participants, allowing further analyses to be performed. In the final data set, less than 0.2% of the data (37 items) was missing, and Little’s MCAR test was not significant, χ ^2^ (1242, *N* = 189) = 1270.23, *p* = .28, indicating that data were missing completely at random. No issues with multicollinearity were present, as evaluated by recommended acceptance limits: tolerance > 0.2 and VIF < 4 [56].

Preliminary bivariate analyses (Table 2) showed no correlation between interpersonal problems (IP) and return to work (RTW), *r* = .03, *p* = .69 or between hostile-dominant interpersonal problems (HDIP) and RTW, *r* = − .08, *p* = .28.

**Table 2:** Correlations for study variables in 189 patients on long-term sick leave

Measure	1		2	3		4		5	6		7		8
1. Age	-												
2. Gender (female)	− 0.03	-											
3. Pain	0.02	0.13			-								
4. Insomnia	0.17^*^	0.01			0.22^**^	-							
5. Fatigue	0.19^**^	0.09			0.10	0.34^**^	-						
6. Anxiety/Depression	0.09	− 0.04			− 0.02	0.45^**^	0.40^**^			-			
7. IP	0.15^*^	0.02			− 0.05	0.38^**^	0.31^**^			0.66^**^	-		
8. HDIP	0.03	− 0.05			− 0.10	0.36^**^	0.21^**^			0.57^**^	0.78^**^	-	
9. RTW	0.12	0.11			− 0.10	0.01	0.01			0.06	0.03	− 0.08	

*Note.* Pain = score on level of somatic pain from the Short-Form Health Status-8. Fatigue = sum score on the Chalder Fatigue Scale. Insomnia = sum score on the Insomnia Severity Index. Anxiety/Depression = sum score on the Hospital Anxiety and Depression Scale. IP = sum score on the Inventory of Interpersonal Problems. HDIP = calculated sub-score from the hostile-dominant quadrant of the Inventory of Interpersonal Problems. RTW (return to work) = average hours worked per week in the first year after rehabilitation

*p < .05

**p < .01

The logistic regression analyses testing predictors of return to work (RTW) are presented in Tables 3 and 4. The first logistic regression model (Table 3) had an acceptable fit to the data, H-L χ ^2^ (8) = 6.38, *p* = .60., but interpersonal problems did not make an independent and statistically significant contribution to the model (OR = 0.82, 95% CI [0.39, 1.72], *p* = .59), rejecting the first hypothesis that more interpersonal problems predict lower likelihood of RTW.

**Table 3:** Summary of the logistic regression analysis testing if interpersonal problems (IP) predicts return to work (RTW) for 189 patients on long-term sick leave, when controlling for symptom levels and demographics

Variable	B	SE	OR	95% CI for OR	
Constant	-1.87	1.15	0.15		
Age	0.03	0.02	1.03	1.00	1.07
Gender (female)	0.72	0.39	2.05	0.95	4.39
Pain	-0.21	0.14	0.81	0.62	1.06
Insomnia	0.00	0.03	1.00	0.95	1.06
Fatigue	-0.03	0.06	0.97	0.86	1.10
Anxiety/depression	0.03	0.03	1.03	0.97	1.09
IP	-0.20	0.38	0.82	0.39	1.72

*Note*. Dependent variable: return to work, dichotomized at more or less than 7.5 h per week on average. Pain = score on level of somatic pain from the Short-Form Health Status-8. Fatigue = sum score on the Chalder Fatigue Scale. Insomnia = sum score on the Insomnia Severity Index. Anxiety/Depression = sum score on the Hospital Anxiety and Depression Scale. IP = sum score on the Inventory of Interpersonal Problems. HDIP = calculated sub-score from the hostile-dominant quadrant of the Inventory of Interpersonal Problems. B = coefficient; SE = standard error; OR = odds ratio; CI = confidence interval.

Pseudo-R^2^ = 0.046 (Cox & Snell) 0.062 (Nagelkerke), PAC = 62%

*p < .05

**Table 4:** Summary of the logistic regression analysis testing if interpersonal hostile-dominant interpersonal problems (HDIP) predicts return to work (RTW) for 189 patients on long-term sick leave, when controlling for symptom levels and demographics

Variable	B	SE	OR	95% CI for OR	
Constant	-1.69	1.16	0.18		
Age	0.28	0.02	1.03	0.99	1.07
Gender (female)	0.70	0.39	2.01	0.93	4.32
Pain	-0.25	0.14	0.78	0.59	1.02
Insomnia	0.10	0.03	1.01	0.95	2.07
Fatigue	-0.03	0.07	0.97	0.85	1.10
Anxiety/depression	0.05	0.03	1.05	0.99	1.11
HDIP	-0.83	0.42	0.44*	0.19	0.98

*Note*. Dependent variable: return to work, dichotomized at more or less than 7.5 h per week on average. Pain = score on level of somatic pain from the Short-Form Health Status-8. Fatigue = sum score on the Chalder Fatigue Scale. Insomnia = sum score on the Insomnia Severity Index. Anxiety/Depression = sum score on the Hospital Anxiety and Depression Scale. IP = sum score on the Inventory of Interpersonal Problems. HDIP = calculated sub-score from the hostile-dominant quadrant of the Inventory of Interpersonal Problems.  B = coefficient; SE = standard error; OR = odds ratio; CI = confidence interval

Pseudo-R^2^ = 0.066 (Cox & Snell) 0.088 (Nagelkerke), PAC = 61%

*p < .05

The second regression (Table 4) had an acceptable fit to the data, H-L χ ^2^ (8) = 4.49, *p* = .81. The model accurately classified 61% of cases and explained between 6.6% (Cox & Snell *R*^2^) and 8.8% (Nagelkerke *R*^2^) of the variance in RTW. In line with our second hypothesis, HDIP made a statistically significant unique contribution to the model, recording an OR of 0.44, 95% CI [0.19, 0.98], p = .045. A one-point higher score in HDIP is associated with a 56% lower chance of successful RTW. These results show that hostile-dominant interpersonal problems predict lower likelihood of RTW in the year following occupational rehabilitation, when controlling for symptom levels.

## Discussion

In line with our predictions (H2), this study found that higher levels of hostile-dominant interpersonal problems (HDIP) in patients on long term sick leave impede a successful return to work (RTW) after occupational rehabilitation. A one-point higher score in HDIP is associated with a 56% lower chance of successful RTW. On the other hand, overall interpersonal problems (IP) was not found to predict RTW (H1). The interpersonal problems of an individual are naturally exacerbated by other health complains, such as chronic pain, fatigue, sleep problems, anxiety and depression. The study therefore controls for these factors, in order to isolate the effect of interpersonal problems that are more stable over time [26].

Interpersonal theory offers suggestions about how dominant and hostile interpersonal problems, respectively, are likely to impede social interaction, cooperation and communication, which are central aspects of the RTW process.

A dominant interpersonal style is motivated by a strong need for agency, for example a need to feel competent, respected and to influence other people. Trying to influence and control others can provoke confrontation and conflict, and is associated with difficulties with authorities. Interacting with superiors is a part of any job, but being on sick leave introduces even more authority figures such as GPs, case workers and other RTW stakeholders. When a dominant person is unsuccessful in imposing the influence or getting the respect they seek, their need is hindered, leading to negative affect such as anger [19].

Hostility and detachment, on the other hand, is rooted in a low need for communion. Getting along with others is simply not a priority, which harms cooperation with both colleagues and RTW stakeholders. In addition, difficulties in feeling close to others and building relationships could lead the returning worker to have less of a support network around them to support through the recovery process.

People who are both hostile and dominant tend to be distrustful and suspicious that others want to exploit them. Even empathic and friendly inquiries from a co-worker or leader could be interpreted as an attempt at manipulation, and hinder social support as well as cooperation.

A comprehensive review by Tjulin and MacEachen [15] found that *preserved dignity, perception of fairness* and *respect for the fact that they still have legitimate health problems* are important social factors at the workplace which influence the RTW process. These factors can be linked to the interpersonal need for agency. Other identified factors include *closeness of relations* and *perceived social support*, which are related to the communion dimension. Note that all these factors are reliant on the perception of the returning worker, and only to a limited extent reflect the actual social landscape of the workplace. Given the suspicious and combative nature of individuals with high HDIP scores, it seems likely that HDIP might impair the RTW process through factors like these. In this way, interpersonal psychology offers a causal explanation for the numerous correlational findings between social factors and RTW.

In psychotherapy research, hostile-dominance has been found to negatively correlate with therapeutic alliance [34, 37], and might therefore impede the therapeutic process in occupational rehabilitation, making these patients benefit less from therapy. Additionally, the concept of alliance, in a broader sense, denotes the degree of trust and cooperation in any relation, including outside of psychotherapy. Poor alliance with colleagues and RTW stakeholders could impede various aspects of the RTW process.

Furthermore, a link has recently been demonstrated between hostile-dominance and the construct *pain catastrophizing*, which is defined as an exaggerated negative orientation to actual or anticipated pain [57]. Ryum et al. [58] showed that HDIP explained unique variance in pain catastrophizing, indicating that the interpersonal context of pain conditions is likely more important than previously acknowledged [58, 59]. In this light, pain catastrophizing can be understood as a strategy these individuals use to communicate their need for validation and support. This could be a useful short-term strategy, but in the longer term it might contribute to both social rejection and exacerbate their pain issues, both affecting RTW negatively.

The results are further illuminated by the lack of support for H1: Overall interpersonal problems did not predict who successfully returned to work. The overall IP score reflects the sum of a variety of maladaptive interpersonal styles, not all of which are equally problematic in the context of returning to work. For example, friendly-submissive interpersonal strategies (such as being overly friendly, submissive and eager to please) are a double-edged sword: These strategies make people exploitable and over-reliant on others – but they also tend to make people very cooperative, a valued trait in employees and co-workers. The same friendly-submissive strategies are also beneficial in psychotherapy, as they are associated with a better therapeutic alliance and better outcome [34, 39]. In an RTW process, the benefits of some interpersonal problems could very well outweigh the personal costs, possibly explaining our null result for H1.

In a short-term therapy setting such as the one described in this study, it is also likely that friendly-submissive traits get more attention, and thus see larger reductions in associated symptoms, than hostile-dominant traits. It is easier for patients to admit (both to themselves and others) to “have difficulties setting boundaries” than “being too suspicious and self-centered”, and therefore also less risky for therapists to address challenges of the first type. This dynamic might partially explain the support for H2, but not H1.

### Strengths and Limitations

This study has several methodological strengths, one of these being the breadth and completeness of collected data. Another is the quality of the RTW measure, using data extracted directly from the database of the Norwegian Health and Welfare Service (NAV). The prospective cohort design and the heterogeneity of the sample (in terms of clinical symptoms and high comorbidity) has several advantages. The design is well suited to explore the influence of interpersonal problems on RTW, without the single-diagnosis limitation many similar studies have [14, 52]. It also gives the study good ecological validity, as the study population is fairly representative of the general sick leave population in Norway.

The study also has limitations worth mentioning. First, in order to achieve sufficient statistical power given the number of predictors, the outcome variable had to be dichotomized. This rules out sensitivity analyses (i.e. using other thresholds than 7.5 h / week) and considerations about trajectories in and out of work, but still yields a clear signal about the importance of HDIP (relative to other predictors) on RTW - which is the main point of the study. Hara [54] writes extensively about the trajectories of our study population.

Second, all clinical measures were assessed by self-report, while the gold standard for evaluating psychiatric symptoms is clinical assessment. However, all questionnaires used (IIP-64, ISI, SF-8, HADS, CFS) are thoroughly validated in different clinical populations.

Third, the sample in this study was relatively small (N = 189), meaning the findings are likely underestimated. A larger sample size would have yielded more statistical power and reduced the risk of Type-2 errors.

Fourth, the full regression - including demographic and symptom variables - explained a rather low proportion (7–10%) of the variance in RTW, indicating that the majority of what influences RTW are factors outside the ones examined in this study. This speaks to the complexity of the RTW construct, a topic covered in detail elsewhere [60].

### Implications and Future Directions

Higher levels of HDIP seem to hinder successful RTW. Put another way, individuals with higher levels of HDIP seem to have particular problems benefitting from traditional RTW approaches, implying that extra attention should be given to this under-studied population. Future research should aim to identify where in the RTW process these traits create problems, as well as which interventions most effectively facilitate RTW for them.

Crucially, interpersonal problems, including dominance, paranoia and hostility, can be reduced in therapy [24, 25, 61]. Several psychotherapy methods focus on reducing dysfunctional personality traits, such as mentalization based therapy, schema therapy and interpersonal therapy [62]. This kind of therapeutic work requires more time and effort than the more widespread symptom-focused therapies, but is likely much more cost-effective in the long term.

Furthermore, acknowledging the role of interpersonal problems yields new perspectives on how to design an effective RTW process, both at the workplace and in the health and welfare sector. Specialized aid in conflict management or tools for facilitating difficult conversations between the worker and employer or co-workers could prove useful. Guidelines, accommodations and training within organizations could also help.

More broadly, future research in the field of occupational rehabilitation should keep individual differences, including personality traits and interpersonal problems, in mind. Individuals with HDIP are of special interest. Specifically, it could be useful to investigate how levels of HDIP affect the quality of the returning worker’s relationships with superiors, coworkers, health care professionals and other stakeholders. Too little is also known about their experience of the RTW process and its different stages. It is our hope that this study can serve as a starting point for a clearer understanding of this problem space.

## Conclusion

To our knowledge, the present study is the first to examine the intersection between interpersonal problems and occupational rehabilitation. The main finding is that higher levels of hostile-dominant interpersonal problems seem to significantly reduce chances of a successful return to work after long-term sick leave. The effect emerges when controlling for subjective health complaints like chronic pain, fatigue, sleep problems, and mild psychological problems. This novel finding clearly demonstrates the importance of considering patients’ interpersonal problems in the field of occupational rehabilitation, and particularly in the context of returning to work after long term sick leave. The results could have major implications for policymakers, employers and healthcare providers alike, and future research should address questions about the nature of the relationship between HDIP and return to work.

## References

[1] OECD. Sickness, disability and work: breaking the barriers: a synthesis of findings across OECD countries. OECD Publishing; 2010.

[2] Mæland S, Werner EL, Rosendal M, Jonsdottir IH, Magnussen LH, Ursin H, et al. Diagnoses of patients with severe subjective health complaints in Scandinavia: a cross sectional study. ISRN Public Health. 2012;2012. 10.3109/02813432.2013.844412 PMC3860299 24164371

[3] Norwegian Health and Welfare Service. Sick leave statistics. 2019, December 5. https://www.nav.no/no/NAV+og+samfunn/Statistikk/Sykefravar+-+statistikk/Sykefravar. Accessed 20.12.19.

[4] Engel GL (1977). The need for a new medical model: a challenge for biomedicine. Science.

[5] Knauf MT, Schultz IZ, Schultz IZ, Gatchel RJ (2016). Current conceptual models of return to work. Handbook of return to work: from research to practice.

[6] Mæland S, Werner EL, Rosendal M, Jonsdottir IH, Magnussen LH, Lie SA (2013). Sick-leave decisions for patients with severe subjective health complaints presenting in primary care: a cross-sectional study in Norway, Sweden, and Denmark. Scand J Prim Health Care.

[7] Hara KW, Borchgrevink PC, Jacobsen HB, Fimland MS, Rise MB, Gismervik S (2018). Transdiagnostic group-based occupational rehabilitation for participants with chronic pain, chronic fatigue and common mental disorders. A feasibility study. Disabil Rehabil.

[8] Hagen R, Johnson SU, Rognan E, Hjemdal O (2012). Towards a common ground: a transdiagnostical approach to psychological treatment. J Norwegian Psychol Assoc.

[9] Cancelliere C, Donovan J, Stochkendahl MJ, Biscardi M, Ammendolia C, Myburgh C (2016). Factors affecting return to work after injury or illness: best evidence synthesis of systematic reviews. Chiropr Man Ther.

[10] Finnes A, Enebrink P, Ghaderi A, Dahl J, Nager A, Öst L-G (2019). Psychological treatments for return to work in individuals on sickness absence due to common mental disorders or musculoskeletal disorders: a systematic review and meta-analysis of randomized-controlled trials. Int Arch Occup Environ Health.

[11] Becker D, Whitley R, Bailey EL, Drake RE (2007). Long-term employment trajectories among participants with severe mental illness in supported employment. Psychiatric Serv.

[12] Rueda S, Chambers L, Wilson M, Mustard C, Rourke SB, Bayoumi A (2012). Association of returning to work with better health in working-aged adults: a systematic review. Am J Public Health.

[13] Kwan HC, Schultz IZ. Work accommodations: a social perspective. Handbook of return to work. Springer; 2016. pp. 271–88.

[14] Henderson M, Harvey SB, Øverland S, Mykletun A, Hotopf M (2011). Work and common psychiatric disorders. J R Soc Med.

[15] Tjulin Å, MacEachen E, Schultz IZ, Gatchel RJ (2016). The importance of Workplace Social Relations in the return to work process: a missing piece in the return to work puzzle?. Handbook of return to work: from research to practice.

[16] Leary T (1957). Interpersonal diagnosis of personality: a functional theory and methodology for personality evaluation.

[17] Horowitz LM (1979). On the cognitive structure of interpersonal problems treated in psychotherapy. J Consult Clin Psychol.

[18] Alden LE, Wiggins JS, Pincus AL (1990). Construction of circumplex scales for the inventory of interpersonal problems. J Pers Assess.

[19] Horowitz LM (2004). Interpersonal foundations of psychopathology.

[20] Bjerke E, Hansen RS, Solbakken OA, Monsen JT (2011). Interpersonal problems among 988 norwegian psychiatric outpatients. A study of pretreatment self-reports. J Compr Psychiatry.

[21] Karterud S, Wilberg T, Urnes Ø. Personality psychiary. 2 ed. Oslo: Gyldendal Akademic Publisher; 2017.

[22] Hengartner MP (2015). The detrimental impact of maladaptive personality on public mental health: a challenge for psychiatric practice. Front Psychiatry.

[23] World Health Organization. International Statistical Classification of Diseases and Related Health Problems. 11 ed. 2019.

[24] McFarquhar T, Luyten P, Fonagy P (2018). Changes in interpersonal problems in the psychotherapeutic treatment of depression as measured by the inventory of interpersonal problems: a systematic review and meta-analysis. J Affect Disord.

[25] Rosenthal RN, Muran JC, Pinsker H, Hellerstein D, Winston A (1999). Interpersonal change in brief supportive psychotherapy. J Psychother Pract Res.

[26] Vittengl JR, Clark LA, Jarrett RB (2003). Interpersonal problems, personality pathology, and social adjustment after cognitive therapy for depression. Psychol Assess.

[27] Østby KA, Czajkowski N, Knudsen GP, Ystrom E, Gjerde LC, Kendler KS (2014). Personality disorders are important risk factors for disability pensioning. Soc Psychiatry Psychiatr Epidemiol.

[28] Schultz IZ, Krupa T, Rogers ES, Schultz IZ, Rogers ES (2011). Best Practices in Accommodating and retaining persons with Mental Health Disabilities at Work: answered and unanswered questions. Work Accommodation and Retention in Mental Health.

[29] Muschalla B, Fay D, Seemann A (2016). Asking for work adjustments or initiating behavioural changes–what makes a ‘problematic co-worker’score brownie points? An experimental study on the reactions towards colleagues with a personality disorder. Psychol health Med.

[30] Bordin ES. The Generalizability of the Psychoanalytic Concept of the Working Alliance. Psychotheraphy: Theory, research and practice. 1979;16(3):252 – 60.

[31] Safran JD, Muran JC (2000). Negotiating the therapeutic alliance: a relational treatment guide.

[32] Martin DJ, Garske JP, Davis MK (2000). Relation of the therapeutic alliance with outcome and other variables: a meta-analytic review. J Consult Clin Psychol.

[33] Castonguay LG, Constantino MJ, Boswell JF, Kraus DR, Horowitz LM, Strack S (2010). The Therapeutic Alliance: Research and Theory. Handbook of interpersonal psychology.

[34] Connolly Gibbons MB, Crits-Christoph P, de la Cruz C, Barber JP, Siqueland L, Gladis M (2003). Pretreatment expectations, interpersonal functioning, and symptoms in the prediction of the therapeutic alliance across supportive-expressive psychotherapy and cognitive therapy. Psychother Res.

[35] Jones DN, Paulhus DL. Differentiating the dark triad within the interpersonal circumplex. In: Horowitz LM, Strack S, editors. Handbook of interpersonal psychology. 2012. p. 249–67.

[36] Ettner SL, Schultz IZ, Rogers ES (2011). Personality disorders and work. Work Accomodation and Retention in Mental Health.

[37] Cookson A, Daffern M, Foley F (2012). Relationship between aggression, interpersonal style, and therapeutic alliance during short-term psychiatric hospitalization. Int J Ment Health Nurs.

[38] Howard I, Turner R, Olkin R, Mohr DC (2006). Therapeutic alliance mediates the relationship between interpersonal problems and depression outcome in a cohort of multiple sclerosis patients. J Clin Psychol.

[39] Muran JC, Segal ZV, Samstag LW, Crawford CE (1994). Patient pretreatment interpersonal problems and therapeutic alliance in short-term cognitive therapy. J Consult Clin Psychol.

[40] de Dreu CK, Van Dierendonck D, Dijkstra MT (2004). Conflict at work and individual well-being. Int J Confl Manage.

[41] Jaramillo F, Mulki JP, Boles JS (2011). Workplace stressors, job attitude, and job behaviors: is interpersonal conflict the missing link?. J Personal Sell Sales Manage.

[42] Holtforth MG, Lutz W, Grawe K (2006). Structure and change of the IIP-D pre-and postpsychotherapy: a replication using a swiss clinical sample. Eur J Psychol Assess.

[43] Fimland MS, Vasseljen O, Gismervik S, Rise MB, Halsteinli V, Jacobsen HB (2014). Occupational rehabilitation programs for musculoskeletal pain and common mental health disorders: study protocol of a randomized controlled trial. BMC Public Health.

[44] Hara KW, Bjørngaard JH, Brage S, Borchgrevink PC, Halsteinli V, Stiles TC (2018). Randomized controlled trial of adding telephone follow-up to an occupational rehabilitation program to increase work participation. J Occup Rehabil.

[45] Ruiz MA, Pincus AL, Borkovec TD, Echemendia RJ, Castonguay LG, Ragusea SA (2004). Validity of the inventory of interpersonal problems for predicting treatment outcome: an investigation with the Pennsylvania Practice Research Network. J Pers Assess.

[46] Monsen JT, Hagtvet KA, Havik OE, Eilertsen DE (2006). Circumplex structure and personality disorder correlates of the interpersonal problems model (IIP-C): construct validity and clinical implications. Psychol Assess.

[47] Zigmond AS, Snaith RP (1983). The hospital anxiety and depression scale. Acta psychiatrica Scandinavica.

[48] Leiknes KA, Dalsbø TK, Siqveland J. Measurement properties of the norwegian version of the hospital anxiety and Depression Scale (HADS). The Norwegian Institute of Public Health; 2016.

[49] Chalder T, Berelowitz G, Pawlikowska T, Watts L, Wessely S, Wright D (1993). Development of a fatigue scale. J Psychosom Res.

[50] Bastien CH, Vallières A, Morin CM (2001). Validation of the Insomnia Severity Index as an outcome measure for insomnia research. Sleep Med.

[51] Landmark T, Romundstad P, Dale O, Borchgrevink PC, Kaasa S (2012). Estimating the prevalence of chronic pain: validation of recall against longitudinal reporting (the HUNT pain study). Pain.

[52] Hara KW, Bjørngaard JH, Jacobsen HB, Borchgrevink PC, Johnsen R, Stiles TC (2018). Biopsychosocial predictors and trajectories of work participation after transdiagnostic occupational rehabilitation of participants with mental and somatic disorders: a cohort study. BMC Public Health.

[53] Clark LA, Watson D (1995). Constructing validity: basic issues in objective scale development. Psychol Assess.

[54] McMillan JH, Schumacher S (2001). Research in Education: a conceptual introduction.

[55] Cohen J (1988). Statistical power analysis for the behavioral sciences.

[56] Hair JF, Anderson RE, Babin BJ, Black WC (2010). Multivariate data analysis: a global perspective.

[57] Sullivan MJ, Thorn B, Haythornthwaite JA, Keefe F, Martin M, Bradley LA (2001). Theoretical perspectives on the relation between catastrophizing and pain. Clin J Pain.

[58] Ryum T, Jacobsen HB, Borchgrevink PC, Landrø NI, Stiles TC (2019). Interpersonal problems as a predictor of pain catastrophizing in patients with chronic pain. Scandinavian J pain.

[59] Boersma K, Flink IK, Linton SJ (2019). Considering the interpersonal context of pain catastrophizing. Scandinavian J Pain.

[60] Loisel P, Buchbinder R, Hazard R, Keller R, Scheel I, Van Tulder M (2005). Prevention of work disability due to musculoskeletal disorders: the challenge of implementing evidence. J Occup Rehabil.

[61] Horowitz LM, Rosenberg SE, Bartholomew K (1993). Interpersonal problems, attachment styles, and outcome in brief dynamic psychotherapy. J Consult Clin Psychol.

[62] Karterud S, Wilberg T, Urnes Ø. Personlighetspsykiatri. 2 ed. Oslo: Gyldendal; 2010.

